# Psychotropic medication use and future unexplained and injurious falls and fracture amongst community-dwelling older people: data from TILDA

**DOI:** 10.1007/s41999-023-00786-x

**Published:** 2023-05-09

**Authors:** Eleanor Gallagher, Mustafa Mehmood, Amanda Lavan, Rose Anne Kenny, Robert Briggs

**Affiliations:** 1 Mercer’s Institute of Successful Ageing, St James’s Hospital, Dublin, Ireland; 2 Discipline of Medical Gerontology, Trinity College Dublin, Dublin, Ireland; 3 The Irish Longitudinal Study on Ageing, Trinity College Dublin, Dublin, Ireland; 4 Department of Emergency Medicine, St James’s Hospital, Dublin, Ireland

**Keywords:** Psychotropic, Falls, Older, Antidepressant, Fractures, Antidepressants

## Abstract

**Purpose:**

Psychotropic medications (antidepressants, anticholinergics, benzodiazepines, ‘Z’-drugs and antipsychotics) are frequently identified as Falls Risk Increasing Drugs. The aim of this study is to clarify the association of psychotropic medication use with future falls/fracture amongst community-dwelling older people.

**Methods:**

Participants ≥ 65 years from TILDA were included and followed from Waves 1 to 5 (8-year follow-up). Incidence of falls (total falls/unexplained/injurious) and fracture was by self-report; unexplained falls were falls not caused by a slip/trip, with no apparent cause. Poisson regression models reporting incidence rate ratios (IRR) assessed the association between medications and future falls/fracture, adjusted for relevant covariates.

**Results:**

Of 2809 participants (mean age 73 years), 15% were taking ≥ 1 psychotropic medication. During follow-up, over half of participants fell, with 1/3 reporting injurious falls, over 1/5 reporting unexplained falls and almost 1/5 reporting fracture. Psychotropic medications were independently associated with falls [IRR 1.15 (95% CI 1.00–1.31)] and unexplained falls [IRR 1.46 (95% CI 1.20–1.78)]. Taking ≥ 2 psychotropic medications was further associated with future fracture (IRR 1.47 (95% CI 1.06–2.05)]. Antidepressants were independently associated with falls [IRR 1.20 (1.00–1.42)] and unexplained falls [IRR 2.12 (95% CI 1.69–2.65)]. Anticholinergics were associated with unexplained falls [IRR 1.53 (95% CI 1.14–2.05)]. ‘Z’-drug and benzodiazepine use were not associated with falls or fractures.

**Conclusion:**

Psychotropic medications, particularly antidepressants and anticholinergic medications, are independently associated with falls and fractures. Regular review of ongoing need for these medications should therefore be central to the comprehensive geriatric assessment.

## Key Summary Points

**Aim:**

To examine the association of psychotropic medication use with future unexplained and injurious falls and fracture in a large cohort of community-dwelling older people.

**Findings:**

Psychotropic medication use was independently associated with falls, unexplained falls and fracture. Antidepressants and anticholinergics were independently associated with future unexplained falls. Benzodiazepines and Z-drugs were not independently associated with falls.

**Message:**

Regular review of ongoing need for these medications should therefore be central to the comprehensive geriatric assessment.

## Introduction

Falls can have a profound impact on the health and general well-being of older people, increasing the risk of significant injury, hospitalisation and early mortality [1, 2]. Importantly falls can also lead to a decline in functional status and loss of independence and can therefore represent a tipping point in terms of admission to a nursing home [3].

Identification of modifiable risk factors for falls is therefore important to inform preventative strategies. One such important modifiable factor is medication use, particularly psychotropic medications. Antidepressants, benzodiazepines, ‘Z’ drugs, anticholinergic medications and antipsychotics are often essential for the management of psychiatric illness and symptoms including mood disturbance, anxiety, psychosis and insomnia amongst older people [4–7]. Exposure to centrally acting medications can impair cognitive function, level of alertness and neuromuscular function however and have been implicated in falls in later life [8, 9]. Many psychotropic medications also impair cardiovascular autonomic reflexes, leading to orthostatic hypotension, an important cause of falls amongst older people [10].

Age-related changes in drug metabolism can lead to higher bioavailability, increasing the potential for drug–drug interactions and for adverse effects of medication use [11]. Despite this, use of psychotropic medication amongst older people in the community [12] and in hospital settings [13] is relatively high, including older people with cognitive impairment [14].

Explained falls are those due to slips or trips [15]. About one-third of falls seen in the emergency department are unexplained [16] with no readily apparent cause, conferring a higher likelihood of admission to hospital [17]. Frequent underlying causes of unexplained falls are orthostatic hypotension [18] and cardiac arrhythmia [19].

Whilst the association between psychotropic medication use and falls is well established, [20, 21] studies to date have not yet examined the link with specific fall types longitudinally and/or robustly adjusted for competing covariates, such as heart disease and chronic disease burden.

The aim of this study therefore is to clarify the longitudinal association between psychotropic medication use and incident falls, injurious falls, unexplained falls and fracture in a large well-described cohort of community-dwelling older people at 8-year follow-up.

## Methods

### Study design

This is a longitudinal cohort study utilising data from The Irish Longitudinal Study on Ageing (TILDA), a large population-based nationally representative sample of community-dwelling older adults aged ≥ 50 years. This study establishes the longitudinal relationship between classes of psychotropic medication use and incident falls and fractures from baseline (Wave 1) to Wave 5 (8-year follow-up).

The TILDA study was designed to investigate how the health, social and economic circumstances of the older Irish population interact in the determination of ‘healthy’ ageing. The TILDA study design has been outlined previously [22]. Briefly, there are three components to data collection: a computer-assisted personal interview carried out by social interviewers in the participants’ own home; a self-completion questionnaire completed and returned by the participant; and a comprehensive centre-based health assessment or a modified home-based health assessment carried out by trained research nurses. Waves of data collection are conducted at 2-yearly intervals, and we used data from Waves 1 to 5, collected between 2009 and 2018.

Participants were included in this study if they were aged ≥ 65 years at Wave 1 and had a medication list examined for medications of interest at baseline and were followed up for at least 2 years, i.e. to Wave 2 of TILDA. Participants were excluded from participation in TILDA at Wave 1 if they had a pre-existing diagnosis of dementia. Subsequent waves were conducted at 2-yearly intervals.

### Psychotropic medications

Medication use was recorded and cross-checked with medication labels at baseline assessment. The Anatomical Therapeutic Chemical (ATC) Classification System was used to identify specific medications. Antidepressants were identified with an ATC code of N06A. Benzodiazepines were identified with an ATC code of N05BA, N05CD and N03AE. ‘Z’ drugs were coded as N05CF and antipsychotics were coded as N05A.

Anticholinergic medications were defined as those classified as having ‘definite’ anticholinergic effects according to the Anticholinergic Cognitive Burden scale [23]. This comprises antidepressants (N06AA, N06AB05), medications acting on the bladder (G04BD), antipsychotics (N05AA01, N05AA03, N05AB03, N05AB06, N05AC02, N05AH03, N05AH04), medications acting on the gastrointestinal system (A03), medications for Parkinson’s Disease (N04A), antihistamines (R06A) and others (M03BA03, M03BC01, N05CM05, N05BB01). Changes to medication use during the follow-up period were not captured.

### Falls and fractures

At Waves 2–5, participants were asked ‘Have you had any falls since the last interview?’ This was used to derive the ‘Falls’ variable.

If they answered yes to this, participants were then asked, ‘Were any of these falls non-accidental, i.e. with no apparent or obvious reason?’ This was used to inform the ‘Unexplained Falls’ variable. Explained falls were therefore accidental falls, due to slips or trips.

Participants were further asked at each wave regarding falls causing injury with ‘Did you injure yourself seriously enough to need medical treatment?’ and this was used to inform the ‘Injurious Falls’ variable.

Participants were also asked about a history of fracture since the last interview (hip, wrist, vertebral and other fractures) at waves 2–5 and this was used to derive the ‘Fractures’ variable.

### Other measures

Highest level of educational attainment was collected by self-report (primary, secondary or tertiary). The Cut Down, Angry, Guilty, Eye Opener (CAGE) scale was used to assess for excess alcohol intake [24]. Heart disease was defined as a self-reported history of heart attack, angina, congestive cardiac failure and/or arrhythmia. Antihypertensive use was defined as being prescribed at least one medication with ATC codes CO2, C03, C07, C08 or C09 after examination of medication list. Chronic disease burden was assessed by self-report of the following conditions: lung disease, eye problems, cancer, osteoporosis, liver disease, arthritis, incontinence, Parkinson’s disease and diabetes. Depressive symptoms were assessed with the Centre for Epidemiological Studies Depression Scale, with a score ≥ 16 indicating clinically significant symptoms [25]. Poor sleep was defined as responding “all the time” when asked how often a participant’s sleep was restless in the last week. Chronic pain was defined as answering ‘severe’ when asked about severity of persistent pain. Self-rated memory problems were defined as answering ‘fair’ or ‘poor’ when asked ‘how would you rate your day-to-day memory at the present time?’

### Statistical analysis

Data were analysed using Stata version 14.1 (Statacorp, Texas).

Baseline characteristics by psychotropic medication use were reported descriptively with differences between groups analysed with chi-square tests and *t* tests.

Poisson regression models, reporting incidence rate ratios (IRR) with 95% confidence intervals, with future falls, unexplained falls, injurious falls and fractures as dependent variables, were used to assess the longitudinal association with psychotropic medications. Two models were used: Model 1 was adjusted only for follow-up time, whilst model 2 was adjusted for age, sex, educational attainment, alcohol excess, heart disease, antihypertensive medication use, chronic disease burden, depressive symptoms, poor sleep, chronic pain and memory problems. These covariates were chosen a priori based on their likelihood of modifying an association with future falls based on prior research studies [26–31].

### Ethics

The TILDA study was approved by the Faculty of Health Sciences Research Ethics Committee at Trinity College Dublin, and all participants gave informed written consent. All experimental procedures adhered to the Declaration of Helsinki. All assessments were carried out by trained research nurses.

## Results

Over 2800 participants were included, and the median follow-up time was 8 years. Baseline characteristics of the study sample by psychotropic medication use are shown in Table 1. Only 18 participants were prescribed antipsychotics, so this cohort was not analysed separately.

**Table 1:** Baseline characteristics by psychotropic medication use

	Total sample *n* = 2809	Antidepressants *n* = 201	Benzodiazepines *n* = 146	‘Z’ drugs *n* = 109	Anticholinergic *n* = 129
Mean age, years (95% CI)	72.9 (72.7–73.1)	73.4 (72.5–74.2)	74.2 (73.2–75.3)	74.1 (72.8–75.4)	73.5 (72.4–74.6)
Age bands					
65–75 years, *n* (%)	1,810 (64)	121 (60)	75 (51)	65 (60)	80 (62)
75–85 years, *n* (%)	848 (30)	69 (34)	62 (42)	31 (28)	41 (32)
≥ 85 years, *n* (%)	151 (5)	11 (5)	9 (6)	13 (12)	8 (5)
Female sex, *n* (%)	1475 (53)*	125 (62)*	101 (69)**	72 (66)*	75 (58)
Educational attainment					
Primary/none, *n* (%)	1166 (42)	92 (46)	73 (50)	51 (47)	55 (43)
Secondary, *n* (%)	947 (34)	66 (33)	46 (34)	28 (26)	46 (36)
Tertiary, *n* (%)	696 (25)	43 (21)	24 (16)	30 (28)	28 (22)
CAGE score					
< 2, *n* (%)	2,257 (80)	148 (74)	111 (76)	86 (79)	98 (76)
≥ 2, *n* (%)	179 (6)	25 (12)	7 (5)	12 (11)	16 (12)
Did not answer, *n* (%)	373 (13)	28 (14)	28 (19)	11 (10)	15 (12)
Heart disease, *n* (%)^A^	629 (22)	62 (31)*	51 (35)**	39 (36)*	41 (32)*
Antihypertensive, *n* (%)^B^	1,558 (55)	127 (63)*	79 (54)	76 (70)*	81 (63)
No. of chronic diseases^C^					
None, *n* (%)	918 (33)	37 (18)**	27 (18)**	22 (20)**	18 (14)**
1, *n* (%)	896 (32)	49 (24)	41 (28)	24 (22)	29 (22)
2–3, *n* (%)	837 (30)	87 (43)	55 (38)	47 (43)	54 (42)
≥ 4, *n* (%)	158 (6)	28 (14)	23 (16)	16 (15)	28 (22)
Depressive symptoms^D^	226 (8)	34 (17)**	43 (29)**	21 (19)**	20 (16)*
Poor sleep^E^	196 (7)	26 (13)*	24 (17)**	16 (15)*	17 (13)*
Chronic pain^F^	251 (9)	29 (14)*	33 (23)**	19 (17)*	20 (16)*
Memory problems^G^	551 (20)	73 (36)**	51 (35)**	33 (30)*	41 (32)**

Chi-square tests used for categorical variables, t tests used for continuous variables

Antidepressants were identified with an ATC code of N06A; Benzodiazepines were identified with an ATC code of N05BA, N05CD and N03AE; Anticholinergics comprised antidepressants with anticholinergic effects (N06AA, N06AB05), medications acting on the bladder (G04BD), antipsychotics with anticholinergic effects (N05AA01, N05AA03, N05AB03, N05AB06, N05AC02, N05AH03, N05AH04), medications acting on the gastrointestinal system (A03), medications for Parkinson’s Disease (N04A), antihistamines (R06A) and others (M03BA03, M03BC01, N05CM05, N05BB01) and ‘Z’ drugs were coded as N05C

*CAGE* cut down, angry, guilty, eye opener scale, *No.* number

^A^Defined as self-report of heart attack, angina, congestive cardiac failure and/or arrhythmia

^B^Prescribed an antihypertensive medication with ATC code of CO2, C03, C07, C08 or C09

^C^Chronic disease burden was assessed by self-report of the following conditions: lung disease, eye problems, cancer, osteoporosis, liver disease, arthritis, incontinence, Parkinson’s disease, and diabetes

^D^Defined as Centre for Epidemiological Studies Depression Scale ≥ 16

^E^Poor sleep defined as reporting restless sleep at least 5 of the last 7 days

^F^Chronic pain defined as reporting severe chronic pain; G memory problems defined a self-report of memory as fair or poor

‘*’ denotes *p* < 0.05; ‘**’ denotes *p* < 0.001

The mean age of the sample at baseline was almost 73 years and 15% (423/2809) were taking at least one psychotropic medication. Participants prescribed psychotropic medication were more likely to be female and had a higher burden of heart disease, chronic illness and depressive symptoms.

**Fig. 1:**
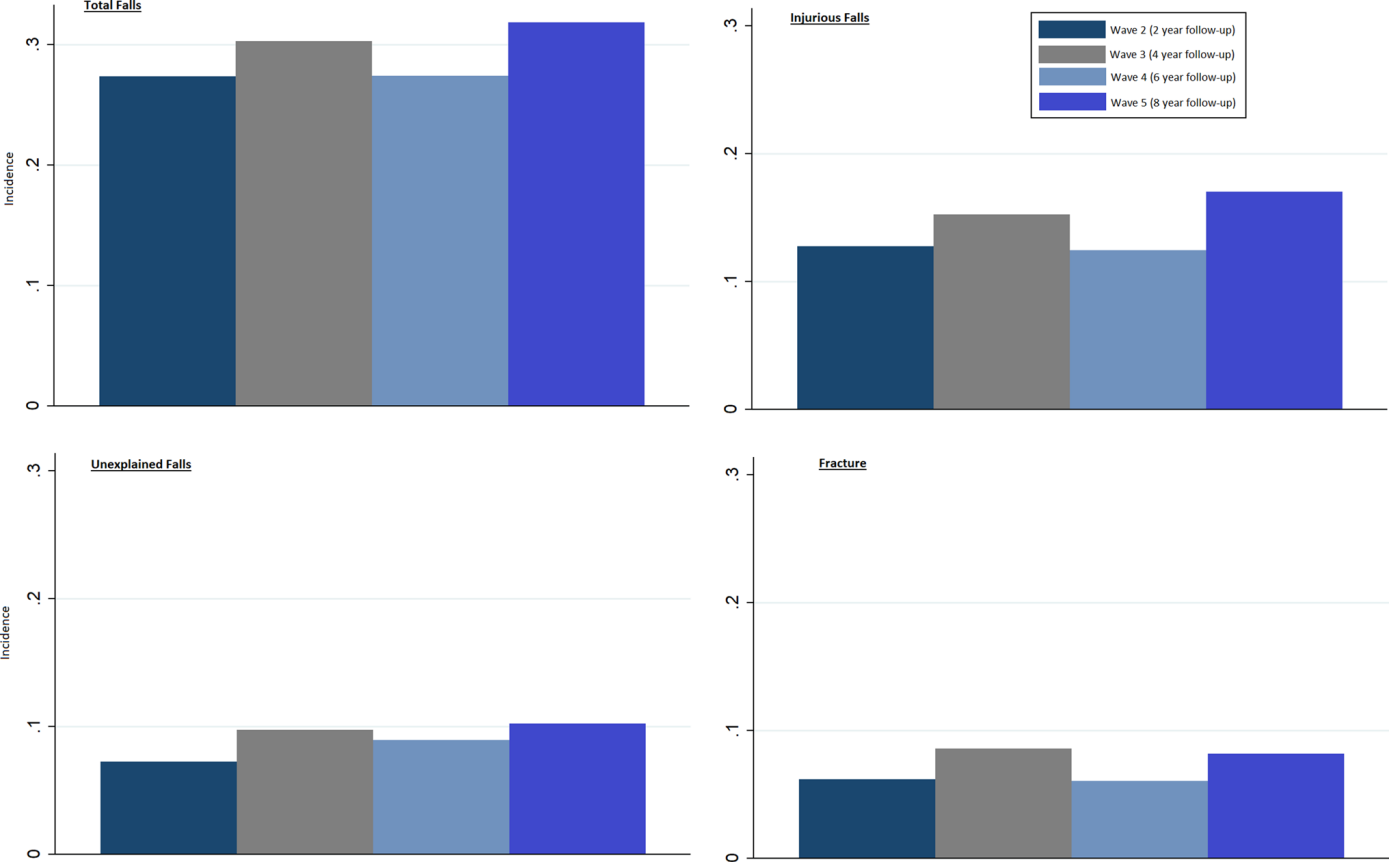
Incidence of falls (total, injurious and unexplained) and fracture during follow-up. At Waves 2–5, participants were asked ‘Have you had any falls since the last interview?’ This was used to derive ‘Total Falls’; Injurious falls were those causing an injury significant enough to require medical attention; Unexplained falls were those not due to a slip or trip and with no apparent cause, collected by self-report; Fracture is hip, wrist, vertebral or other fracture, by self-report

Over half (1548/2809, 55%) of participants had a fall during follow-up, with one-third (904/2809, 32%) reporting an injurious fall, over one-fifth (614/2809, 22%) reporting an unexplained fall and almost one-fifth (520/2809, 19%) reporting a fracture. Incidence of falls and fracture at each Wave is shown in Fig. 1.

### Psychotropic medication and falls

Over 10% (286/2809) of the study sample was taking one psychotropic medication, i.e. either an antidepressant, benzodiazepine, ‘Z’ drug, anticholinergic or antipsychotic, whilst 5% (137/2809) was taking more than one agent.

Psychotropic medication use was independently associated with falls [IRR 1.15 (95% CI 1.00–1.31); *p* = 0.047], unexplained falls [IRR 1.46 (95% CI 1.20–1.78); *p* < 0.001] longitudinally.

**Fig. 2:**
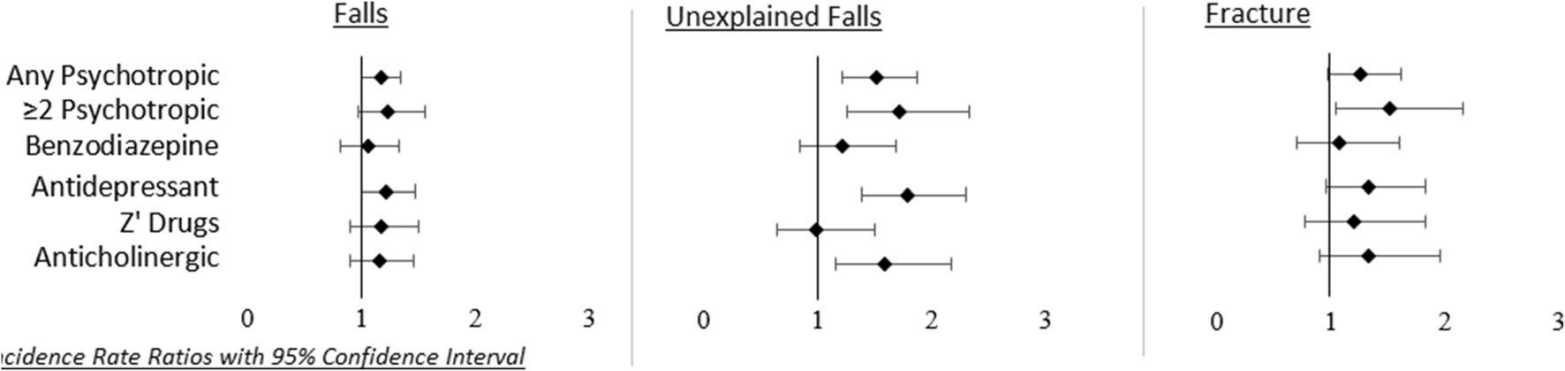
Incidence rate ratios with 95% confidence intervals for association between psychotropic medication use and future falls (total/unexplained) and fracture. Data presented are incidence rate ratios with 95% confidence intervals from fully adjusted models with Injurious and Unexplained falls and Fractures as dependant variables. Models were adjusted for age, sex, educational attainment, alcohol excess, heart disease, chronic disease burden, depressive symptoms, poor sleep, chronic pain, and self-rated memory problems. Fracture comprises hip, wrist, vertebral and other fractures. Antidepressants were identified with an ATC code of N06A; benzodiazepines were identified with an ATC code of N05BA, N05CD and N03AE; anticholinergics comprised antidepressants with anticholinergic effects (N06AA, N06AB05), medications acting on the bladder (G04BD), antipsychotics with anticholinergic effects (N05AA01, N05AA03, N05AB03, N05AB06, N05AC02, N05AH03, N05AH04), medications acting on the gastrointestinal system (A03), medications for Parkinson’s Disease (N04A), antihistamines (R06A) and others (M03BA03, M03BC01, N05CM05, N05BB01) and ‘Z’ drugs were coded as N05C

As shown in Table 2 and Fig. 2, taking more than one psychotropic medication was more closely associated with injurious and unexplained falls, as well as future fracture (IRR 1.47 (95% CI 1.06–2.05); p 0.021) during follow-up. There was a graded relationship between psychotropic medication use and unexplained and injurious falls, as well as fracture.

**Table 2:** Poisson regression models reporting incidence rate ratios with falls/fracture as dependent variables

	Falls		Injurious falls		Unexplained falls		Fracture	
	Unadjusted	Adjusted	Unadjusted	Adjusted	Unadjusted	Adjusted	Unadjusted	Adjusted
Any Psych	IRR 1.30 (1.14, 1.48) *p* < 0.001	IRR 1.15 (1.00, 1.31) *p* = 0.047	IRR 1.34 (1.13, 1.58) *p* 0.001	IRR 1.10 (0.92, 1.31) *p* 0.288	IRR 1.85 (1.54, 2.23) *p* < 0.001	IRR 1.46 (1.20, 1.78) *p* < 0.001	IRR 1.53 (1.24, 1.90) *p* < 0.001	IRR 1.25 (0.99, 1.56) *p* 0.056
≥ 2 Psych	IRR 1.38 (1.13, 1.70) *p* 0.002	IRR 1.21 (0.98, 1.50) *p* 0.070	IRR 1.59 (1.24, 2.05) *p* < 0.001	IRR 1.30 (1.00, 1.68) *p* 0.049	IRR 2.13 (1.62, 2.80) *p* < 0.001	IRR 1.64 (1.23, 2.19) *p* 0.001	IRR 1.80 (1.31, 2.48) *p* < 0.001	IRR 1.47 (1.06, 2.05) *p* 0.021
Benzo	IRR 1.21 (0.98, 1.49) *p* 0.082	IRR 1.05 (0.84, 1.30) *p* 0.694	IRR 1.30 (0.99, 1.70) *p* 0.059	IRR 1.04 (0.79, 1.38) *p* 0.775	IRR 1.60 (1.18, 2.15) *p* 0.002	IRR 1.19 (0.86, 1.62) *p* 0.284	IRR 1.43 (1.01, 2.02) *p* 0.041	IRR 1.08 (0.75, 1.55) *p* 0.680
Antidep	IRR 1.34 (1.13, 1.59) *p* 0.001	IRR 1.20 (1.00, 1.42) *p* = 0.044	IRR 1.44 (1.16, 1.79) *p* < 0.001	IRR 1.22 (0.98, 1.53) *p* 0.079	IRR 2.12 (1.69, 2.65) *p* < 0.001	IRR 1.71 (1.35, 2.16) *p* < 0.001	IRR 1.54 (1.16, 2.03) *p* 0.003	IRR 1.31 (0.98, 1.75) *p* 0.067
‘Z’ drugs	IRR 1.31 (1.04, 1.65) *p* 0.022	IRR 1.15 (0.91, 1.45) *p* 0.251	IRR 1.33 (0.98, 1.80) *p* 0.064	IRR 1.09 (0.80,1.48) *p* 0.576	IRR 1.27 (0.88, 1.85) *p* 0.208	IRR 0.99 (0.68, 1.45) *p* 0.967	IRR 1.48 (1.01, 2.17) *p* 0.043	IRR 1.20 (0.81, 1.76) *p* 0.367
Antichol	IRR 1.26 (1.02, 1.56) *p* 0.032	IRR 1.14 (0.91, 1.41) *p* 0.255	IRR 1.31 (1.00, 1.72) *p* 0.054	IRR 1.13 (0.85, 1.49) *p* 0.402	IRR 1.90 (1.43, 2.52) *p* < 0.001	IRR 1.53 (1.14, 2.05) *p* 0.004	IRR 1.50 (1.07, 2.12) *p* 0.020	IRR 1.31 (0.92, 1.87) *p* 0.128

Antidepressants were identified with an ATC code of N06A; Benzodiazepines were identified with an ATC code of N05BA, N05CD and N03AE; Anticholinergics comprised antidepressants with anticholinergic effects (N06AA, N06AB05), medications acting on the bladder (G04BD), antipsychotics with anticholinergic effects (N05AA01, N05AA03, N05AB03, N05AB06, N05AC02, N05AH03, N05AH04), medications acting on the gastrointestinal system (A03), medications for Parkinson’s Disease (N04A), antihistamines (R06A) and others (M03BA03, M03BC01, N05CM05, N05BB01) and ‘Z’ drugs were coded as N05C

Fracture comprises hip, wrist, vertebral and other fractures

Poisson regression models, reporting incidence rate ratios and 95% confidence intervals, with falls, unexplained falls, injurious falls, and fractures during follow-up as dependent variables, were used to assess the longitudinal association with psychotropic medications. Two models were used: Model 1 was adjusted only for follow-up time, whilst model 2 was adjusted for age, sex, educational attainment, alcohol excess, heart disease, chronic disease burden, depressive symptoms, poor sleep, chronic pain and self-rated memory problems

*Psych* psychotropic medication, *Benzo* benzodiazepine, *Antidep* antidepressant, *Antichol* anticholinergic medication, *IRR* incidence rate ratio

### Antidepressants

Antidepressant use was independently associated with future falls [IRR 1.20 (95% CI 1.00–1.42); *p* = 0.044] and unexplained falls [IRR 1.64 (95% CI 1.23–2.19); *p* = 0.001]. See Table 2 and Fig. 1.

### Benzodiazepines

Benzodiazepine use was not associated with falls, injurious falls, unexplained falls or fracture in fully adjusted models. See Table 2 and Fig. 2.

### ‘Z’ drugs

The use of ‘Z’ drugs was not associated with falls, injurious falls, unexplained falls or fracture in fully adjusted models. See Table 2 and Fig. 2.

### Anticholinergics

Anticholinergic use was associated with an increased likelihood of unexplained falls [IRR 1.50 (95% CI 1.14–2.05); *p* = 0.004] in fully adjusted models. The association with injurious falls was non-significant, as was the association with future fracture.

## Discussion

This study examines the association between psychotropic medication use and future falls and fracture in a cohort of community-dwelling people aged ≥ 65 years at 8-year follow-up. We have shown that taking a single psychotropic medication is independently associated with an almost 50% higher likelihood of unexplained falls, whilst taking more than one psychotropic medication is associated with a 64% increased risk of unexplained falls in fully adjusted models. Psychotropic medication use was associated with falls, unexplained falls and incident fracture, with a closer association with taking more than one psychotropic medication for each of these outcomes.

Prior studies have shown that psychotropic medications increase the risk of falls [20, 21] and fracture [32]. This study adds to the existing evidence base however by robustly controlling for covariates including heart disease and chronic disease burden, over a prolonged follow-up period of 8 years. Further, we also examine the risk associated specifically with unexplained falls, which are more often underpinned by orthostatic blood pressure changes and heart rhythm disturbance, both of which exacerbated by psychotropic medications.

When examined by classes of psychotropic medication, we found that in fully adjusted models, antidepressants and anticholinergic medication were associated with unexplained falls. Perhaps surprisingly benzodiazepine and z-drug use was not associated with falls or fracture in fully adjusted models.

Whilst depression has been shown to increase falls risk amongst older people [28], the strong association between antidepressant use and unexplained falls persisted after adjustment for depressive symptoms burden in this study. TILDA data have previously shown the link between antidepressant use and falls at 4-year follow-up—yet did not examine the longitudinal relationship with fracture [33]. Further, data from TILDA have also demonstrated that antidepressant users had doubled the risk of orthostatic hypotension compared to non-users [34] whilst delayed postural BP stabilisation related to antidepressant use has also been delineated in a cohort of patients with Alzheimer’s disease [35]. Antidepressant use may also accelerate post-menopausal bone loss, contributing to risk of fracture [36].

Our findings regarding ‘Z’ drug are in line with a recent meta-analysis of 14 studies which showed a trend towards increased falls only [37]. Consistent with our findings, anticholinergic medications have been consistently linked with incident falls in later life [38, 39], and we have also demonstrated a strong relationship with unexplained falls in this study, likely also related to effects on cognition, cardiovascular reflexes and gait.

Whilst cross-sectionally benzodiazepines are associated with falls amongst the TILDA cohort [40], we found no significant longitudinal relationship with falls, including injurious and unexplained falls, and fracture in this study. This contrasts with findings from systemic reviews and meta-analyses, which have shown an increased falls risk related to benzodiazepine use [20, 41], but it is important to note that many studies to date have not adjusted for the full spectrum of competing covariates, especially when we consider that benzodiazepines are more often prescribed for people with pre-existing risk factors for fall [42].

There are some limitations of this study which should be noted. Falls outcomes and several covariates (including cardiovascular disease, educational attainment, chronic disease burden, etc.) were by self-report only and could therefore be subject to recall bias, which could impact findings. Further, neuroleptic medication use was captured at baseline, but changes in medication use over time were not captured in the analysis. We could not perform any meaningful analysis on participants taking antipsychotic medications due to the small numbers involved. We also did not have any information on compliance with medications, but data was collected by manually checking participant medication lists. Whilst we have adjusted for a wide range of covariates in this study, the complex and often multifactorial nature of falls amongst older people is such that there remains a possibility of residual confounding that we have not adjusted for. The strengths of this study include the large, well-described population-representative sample of older people, with robust 8-year follow-up. To our knowledge, this is the first study to examine the relationship between psychotropic medication use and unexplained falls amongst older people.

Our study suggests that withdrawal of psychotropic medication use may reduce the risk of falls. This is supported by intervention studies involving small participant numbers [43, 44]. Tool such as STOPPFall, developed to aid de-prescribing of medications that increase falls risk, may help as it involves mostly psychotropic medications [45]. However, de-prescribing psychotropic medications can be challenging in practice even when supported by tools such as this [46]. For example, there is lack of consensus as to when antidepressants should be discontinued in patients with stable late-life depression [47]. Additionally, even though withdrawing of benzodiazepines in older people does not generally lead to significant sleep disturbance [48], it can also be complex and is considered arduous and time-intensive by physicians [49, 50].

In conclusion, we have shown that psychotropic medication use amongst community-dwelling older people significantly increases the likelihood of unexplained falls, including those causing fracture. As part of a comprehensive multifactorial falls assessment, described in recently published World Falls Guidelines [51], de-prescribing of psychotropic medications to reduce falls risk, particularly amongst older people taking more than one agent, should be considered where appropriate and feasible and can be supported by structured de-prescribing tools. If psychotropic medications cannot be discontinued, close attention should be paid to orthostatic BP and ECG monitoring, as well as review of other co-prescribed culprit medications. Further work is required however, in establishing how and when psychotropic medications can be dose-reduced or discontinued.
